# Diversity of *Lactobacillus* Species of Stilton Cheese Relates to Site of Isolation

**DOI:** 10.3389/fmicb.2020.00904

**Published:** 2020-05-12

**Authors:** Diriisa Mugampoza, Konstantinos Gkatzionis, Benjamin M. C. Swift, Catherine E. D. Rees, Christine E. R. Dodd

**Affiliations:** ^1^School of Biosciences, University of Nottingham, Nottingham, United Kingdom; ^2^Department of Food Technology, Kyambogo University, Kampala, Uganda; ^3^Department of Food Science and Nutrition, School of the Environment, University of the Aegean, Lemnos, Greece; ^4^Pathobiology and Population Sciences, Royal Veterinary College, Hertfordshire, United Kingdom

**Keywords:** Stilton cheese, *Lactobacillus*, PFGE, antimicrobial activity, stress tolerance, plantaricin EF genes

## Abstract

This study has characterized the dominant non-starter *Lactobacillus* species isolated from different sites in a Stilton cheese to establish its diversity, stress-tolerance, anti-microbial activity and potential contribution to quality of cheese. Fifty-nine *Lactobacillus* isolates were cultured from the outer crust, blue veins and white core of the cheese and were speciated phenotypically and by 16S rDNA sequence analysis. *Lactobacillus plantarum* was the dominant species detected with only two isolates identified as *Lactobacillus brevis*. Strains were typed by pulse-field gel electrophoresis (PFGE) using the enzyme *Not*I to examine their genomic diversity. Cluster analysis of PFGE patterns produced five major clusters which associated isolates with their sites of isolation within the cheese. One *L. plantarum* isolate from each cheese site was selected and evaluated for salt, acid, relative humidity, and heat tolerance to determine whether stress conditions within the isolation site selected their phenotype. *D*_72°C_ values were 6, 13, and 17 s for strains from the crust, veins and core, respectively, suggesting strains on the crust may not have been able to survive pasteurization and therefore had been added post-pasteurization. All strains recovered from heat injury within 24–48 h at 4°C. pH values of 3, 3.5, and 4 suppressed growth but strains showed a varying ability to grow at pH 4.5 and 5; isolates from the core (which has the lowest pH) were the most acid-tolerant. All strains grew at 3.5 and 5% salt but were suppressed at 10%; those from the crust (which has a lower water activity) were the most halo-tolerant, growing at 8% salt whereas strains from the core were sensitive to this salt concentration. All 57 *L. plantarum* isolates were examined for antimicrobial activity and variable activity against *Lactobacillus pentosus* and other genera was demonstrated; plantaricin EF genes were present in 65% of strains. It was concluded that there are varied phenotypes and genotypes of *Lactobacillus* in a Stilton cheese according to site of isolation. Occurrence of different *L. plantarum* genotypes could contribute to variation in the cheese quality from batch to batch and provides criteria for selecting isolates as potential adjunct cultures.

## Introduction

Stilton is a protected designation of origin (PDO) mold-ripened semisoft blue-veined cheese made from pasteurized cows’ milk in the United Kingdom. Acidification is achieved by the addition of *Lactococcus lactis* as a starter culture, while ripening is promoted by development of the mold *Penicillium roqueforti* as well as non-starter bacteria (Ercolini et al., 2003) and yeasts (Gkatzionis et al., 2014). Microbial communities in Stilton differentiate according to their spatial distribution in the outer crust, blue veins, and white core of the cheese (Ercolini et al., 2003; Gkatzionis et al., 2014). For example, *Kluyveromyces lactis* and *Debaryomyces hansenii* were found to be the dominant yeasts in the blue veins whereas *Candida catenulata* and *Yarrowia lipolytica* were mostly found in the outer crust and white core (Gkatzionis et al., 2014). This diversity in the microbial composition according to spatial distribution was associated with differences in aroma development in each section of the cheese (Gkatzionis et al., 2009, 2013; Price et al., 2013). The bacterial community structure in these cheese sites was also found to be different in each section, comprising *L. lactis*, *Enterococcus faecalis*, *Lactobacillus plantarum*, *Lactobacillus curvatus*, *Leuconostoc mesenteroides*, *Staphylococcus equorum*, and *Staphylococcus* sp., with *L. plantarum* being the dominant species (Hiscox et al., 1940; Sharpe and Brindley, 1956; Whitley, 2002; Ercolini et al., 2003). This complex microbiota is responsible for ripening leading to the typical aroma development of the cheese, although the composition is fortuitous, through accidental introduction during production, and not directly controlled by the producer. It is, thus, evident that the structure and composition of the microbiota of Stilton cheese may reflect natural selection at the different cheese sites due to varying gradients of redox potential, acid, osmotic and other stresses which constitute a selection criterion for these organisms through competition and symbiotic interactions (Turroni et al., 2009). It may also reflect the source of introduction of each isolate.

Whereas the spatial differentiation of Stilton microbial communities has been established at the species level, there is a lack of information on biotypes at subspecies and strain levels. Moreover, individual strains often pose profound effects on cheese flavor and texture characteristics (Broadbent et al., 2003), defects and production inconsistencies. In the present study, the subspecies diversity of the lactobacilli occurring in a Stilton cheese was determined on the basis of their pulse-field gel electrophoresis (PFGE) patterns, morphological and biochemical characteristics, antimicrobial activity, presence and expression of plantaricin genes, as well as response to different stresses typical of the cheese microenvironment.

## Materials and Methods

### Microbial Isolation and Identification

An 8 kg commercial sample of Stilton cheese at the end of ripening (45 days) was purchased from a local retailer in Nottingham city and precisely partitioned into different sections, the outer crust, blue veins and white core. Microbiological analysis was performed on each of the cheese sections as described by Gkatzionis et al. (2014) by aseptically scrapping 130–190 mg micro-samples into sterile o-ringed micro-centrifuge vials (BioSpec Products, Sanford, United Kingdom). The samples were mixed with nine parts of maximum recovery diluent (MRD; Oxoid, Basingstoke, United Kingdom) and four glass beads (2 mm, acid washed, BioSpec Products, Sanford, United Kingdom), and homogenized using a Mini Beadbeater-1 (BioSpec Products) at 2,500 rpm for 2 s × 40 s, cooling on ice between each treatment. Samples of the same cheese section were pooled and 1 ml volumes used for further 10-fold serial dilutions and subsequently plated on M17 agar (Oxoid) and standard Rogosa agar (pH 5.4 ± 0.2 at 25°C, Oxoid). Plates were incubated aerobically/anaerobically in sealed plastic containers enclosed with 2.5 L Anaerogen (AN0035, Oxoid) gas generating sachets for 24–48 h at 30–37°C. After incubation, three to five colonies with different morphologies (large cream-whitish, profuse and small, and elliptical off-white) were randomly selected and streaked twice on Rogosa and M17 agar for purification. The isolates were stored in brain heart infusion (BHI) broth (Oxoid) with the addition of 20% (v/v) glycerol (G/0650/17, Fisher Scientific) at −80°C in a freezer (U570, New Brunswick Scientific, England) until use. Lactic acid bacterial (LAB) isolates were recovered by culturing on Rogosa and M17 agar after incubation for 48 h at 30°C anaerobically and tested for Gram reaction, catalase, oxidase, and motility (Collins et al., 2004). Cell morphology was examined under oil immersion microscopy (Collins et al., 2004).

Identification at species level was achieved by 16S rDNA sequence analysis using *Lactobacillus*-specific V6–V8 primers Lac1 (5′-AGCAGTAGGGAATCTTCCA-3′) and Lac2 (5′-ATTTCACCGCTACACATG-3′) adopted from Lopez et al. (2003). Genomic DNA was extracted as described by Pitcher et al. (1989). PCR conditions were adopted from Lopez et al. (2003). Speciation of *L. plantarum* was validated using *rec* A gene multiplex PCR analysis as described by Torriani et al. (2001).

### Physiological Characterization

Metabolism of various carbon sources for the 59 *Lactobacillus* isolates was determined using API 50 CHL (BioMerieux, France). Profiles of the biochemical reactions were recorded visually, and identifications obtained using the API software (BioMerieux).

### Subspecies Typing by PFGE

For each isolate, a colony was inoculated in 10 ml MRS broth (Oxoid) and incubated for 16 h at 30°C. The culture (1.5–3 ml) was centrifuged for 1 min at 13,000 *g* to harvest the cells. Genomic DNA was prepared *in situ* in agarose blocks as described by Moore and Datta (1994) and Yeung et al. (2004). Genomic DNA was digested with 40 U of *Not*I (Promega, Southampton, United Kingdom), which recognizes G + C rich sequences, and produced suitable PFGE patterns for the tested isolates (Yeung et al., 2004). This is due to relatively low guanine and cytosine content (35–47 G + C mol %) in the genome of lactobacilli. Gel electrophoresis was performed using the method of Vancanneyt et al. (2006) with minor modifications. Plug slices were loaded into wells of 1% (w/v) PFGE certified agarose (Bio-Rad, Philadelphia, PA, United States) gel prepared in 0.5X TBE buffer containing 100 μM thiourea (Sigma). Electrophoresis was performed in 2 L of 0.5X TBE buffer containing 100 μM thiourea in a contour-clamped homogenous electrophoresis cell (CHEF) DRII (Bio-Rad) at 14°C for 21 h at 6 V/cm and pulse times ramping linearly from 4 to 45 s at a switching angle of 120° and pump pressure of 80 rpm. A 50–1,000 kbp DNA ladder (Sigma) was used as a molecular size marker. After electrophoresis, the gel was stained in 100 ml of sterile deionized water (SDW) containing 10 μl (10 mg/ml) of ethidium bromide (Fisher Scientific) for 1 h at room temperature, and subsequently de-stained in 100 ml of SDW for 30 min at room temperature. Images were visualized on a UV *trans*-illuminator (Bio-Rad) and recorded with Quantity one Gel Doc software (Bio-Rad).

### Cluster Analysis

The PFGE patterns were normalized and processed using FP Quest software (Bio-Rad) to generate the dendrogram. Clustering was calculated using the un-weighted pair group method with arithmetic averages (UPGMA) and comparison of the patterns done using the band-based Dice similarity coefficient. Clusters were defined at a similarity level of 52% (Tenover et al., 1995). The significance of clustering was tested using analysis of molecular variance (Excoffier et al., 1992) by calculating the PhiPT value (Φ*_*PT*_*), a measure of sub-population or cluster genetic differentiation that suppresses intra-individual variation. Calculation of Φ*_*PT*_* was performed using GenAlEx v.6.5 software.

### Acid and Salt Tolerance

Acid and salt tolerance of selected *L. plantarum* isolates was evaluated using the approach of Succi et al. (2005) and Pelinescu et al. (2009). Isolates were grown in 10 ml MRS broth (Oxoid) overnight at 30°C. The culture was transferred into 30 ml of MRS broth acidified to pH 3, 3.5, 4, 4.5, and 5 using lactic acid (Thermo Fisher Scientific, Basingstoke, United Kingdom) to attain an inoculum level of 10^5^ CFU ml^–1^. The broths were incubated at 30°C and 100 rev min^–1^ in a shaking incubator (Gallenkamp, Loughborough, United Kingdom). Aliquots (0.1 ml) were withdrawn at different time intervals; these were used for enumeration of the injured and un-injured viable counts on the non-selective BHI agar (Oxoid) at 30°C, anaerobically. Salt tolerance was examined under the same conditions by inoculating the isolates into MRS broth containing 3.5, 5, 8, and 10% sodium chloride (Fisher Scientific, United Kingdom).

### Desiccation Tolerance

Desiccation tolerance was determined according to the method of Pedersen et al. (2008). Cells from 1 ml MRS broth (Oxoid) culture were harvested by spinning at 9,000 *g* for 2 min, 4°C and suspended in 1 ml of maximum recovery diluent (MRD, Oxoid). Aliquots (50 μl) were added to two sets of U-shaped wells in 96 mm × 128 mm micro-titer plates (Thermo Fisher Scientific, Waltham, MA, United States). Un-inoculated wells were filled with 50 μl sterile MRD (blank). The plates were dried for 24 h at 30°C and subsequently transferred into separate desiccators with RH established and maintained at 33 ± 1 and 54 ± 1% using saturated solutions of magnesium chloride hexahydrate (Acros Organics, NJ, United States) and magnesium nitrate hexahydrate (Sigma-Aldrich, MO, United States), respectively. The desiccators were incubated at 20 ± 1°C for 7 days. Desiccated cells were rehydrated for 30 min by adding 0.1 ml MRD, and then mixed by pipetting. Then, 0.1 ml of each sample was mixed with 0.9 ml MRD to obtain the initial dilution of 10^–1^. Further 10-fold dilutions up to 10^–9^ were prepared in MRD. Each sample was plated in triplicate on BHI agar and incubated anaerobically at 30°C for 48 h. Plates with 30–300 colonies were enumerated to obtain counts at different time points. In order to ascertain the behavior of the isolates in the absence of nutrients, the experiment was repeated by suspending the cells in sterile deionized water (Fermentas, Altrincham, United Kingdom) prior to drying and desiccation.

### Heat Tolerance and Recovery of Injured Cells

Heat tolerance was ascertained in sterile cows’ milk (3% fat; Drinks Brokers Ltd., Norfolk, England). *L. plantarum* isolates were cultivated in MRS broth (Oxoid) at 30°C for 24 h. Cells were harvested from 1 ml culture by centrifugation at 13,000 *g*, 1 min and washed in phosphate buffered saline (PBS, Oxoid). The cell pellet was re-suspended in 1 ml PBS and spiked into milk at a final concentration of 9 log_10_ CFU ml^–1^. Inoculated milk was dispensed in 0.5 ml aliquots into 1.5 ml Wheaton vials (Fisher Scientific, Suwanee, GA, United States). The vials were fully immersed in a water bath (Grant Instruments, Royston, United Kingdom) pre-heated to 72 ± 1°C, and 0.1 ml samples withdrawn at different time points into pre-cooled (4°C) MRD (Oxoid). Each cooled sample was serially diluted in MRD and the dilutions (0.1 ml) were plated in duplicate onto BHI (Oxoid) and MRS agar (Oxoid) for enumeration of the viable counts. The counts were normalized to log_10_ CFU ml^–1^, converted into percent survivors and plotted against time (s). Viable counts below the 1 log_10_ CFU ml^–1^ limit of detection were regarded as negative recovery. A *D*_72°C_ value for each isolate was calculated from the linear portion of the curve (0–50 s) with a correlation coefficient, *r*^2^, >0.90 (Ahmed et al., 1995). Recovery from heat stress was assessed by enumeration of viable counts on MRS and BHI agar after keeping the heat-treated milk samples for 48 h at 4°C. Milk inoculated with different levels of each of the isolates and kept for 48 h at 4°C was used as a growth control for the heat recovery experiments (Swift, 2010; Mugampoza, 2013).

### Antimicrobial Activity of the Isolates and Screening for Bacteriocin Genes

Screening for bacteriocin-producing *Lactobacillus* isolates was carried out on MRS agar (Oxoid) using the plate agar overlay method of Powell et al. (2007). All plates were incubated at 30°C for 24 h. The plates with 30–100 colonies were overlaid with a layer of 0.7% (w/v) BHI agar (Oxoid) seeded with 10^6^ CFU ml^–1^ of the indicator strain. The seeded plates were incubated at 37°C for 24 h. *Lactobacillus* colonies with the largest zones of growth inhibition measured using a hand ruler were isolated, purified and used for confirmation of antimicrobial activity.

Antimicrobial activity was confirmed by using the paper diffusion assay (Albano et al., 2007) using *Pseudomonas aeruginosa* glaxo 3, *Escherichia coli* 0157: H7-*stx*, *Listeria monocytogenes* NCTC 11944, and *Lactobacillus pentosus* NCIMB 8026 which were the most sensitive strains from the agar overlay assays. *Lactobacillus* isolates were grown in 10 ml MRS broth (Oxoid) for 24 h at 30°C and 100 rev min^–1^. The culture was centrifuged at 3,400 *g* (Megafuge 40R, Thermo Fisher Scientific) for 15 min at 4°C to obtain a cell-free supernatant (CFS). CFS was sterilized with a 0.2 μm membrane filter (Minisart Sartorius, Göttingen, Germany) and stored at 4°C until use. Indicator strains were grown at 30–37°C in 10 ml BHI broth depending on the optimum temperature of each strain. Petri dishes filled with 20 ml of 1.5% (w/v) MRS agar were overlaid with 10 ml of BHI or MRS with 0.7% (w/v) agar, as appropriate, inoculated with 10^6^ CFU ml^–1^ indicator strain. Sterile filter paper discs (Whatman AA, 13 mm, Fisher Scientific) were soaked in the CFS for 30–60 min and then applied to the seeded plates in duplicate. All plates were incubated for 24 h at 37°C and the diameter (mm) of the resulting zone of inhibition measured from the edge of the paper disc to the edge of the zone of clearing. Halos extending for 0.5 mm or more were considered as positive. The nature of antimicrobial activity was examined using CFS treated with 1N NaOH to bring the pH to 6.5–7 to neutralize acids and/or with 500 U ml^–1^ catalase (Sigma) to eliminate hydrogen peroxide. Treatment with 1 mg ml^–1^ proteinase K (Fermentas) was used to ascertain whether activity was due to bacteriocin (putative plantaricin) production. Enzyme treated supernatants were incubated for 30 min 37°C and the enzymes inactivated by boiling for 2 min. Presence of the various plantaricin genes was examined using PCR as described by Mugampoza 2013, (Section 3.3.4.3); the primers and PCR conditions were adopted from Cho et al. (2010) and Yi et al. (2010). A pediocin-producing strain of *Pediococcus acidilactici* NCIMB 700993 was used as a control.

### Statistical Analysis of Count Data

Challenge experiments were performed in triplicate and counts were normalized by conversion to log_10_ CFU ml^–1^. Single factor ANOVA computed with the Predictive Analytical Software, v.17 was used to determine the statistical significance of the data. Results with *p-*values less than 0.05 were considered significant. Viable counts below the 1 log_10_ CFU ml^–1^ limit of detection were regarded as negative.

## Results

### Microbial Isolation and Identification

Based on colony characteristics, cell morphologies and biochemical tests, 61 *Lactobacillus* isolates (Supplementary Table S1) were recovered from different sections of the Stilton cheese on Rogosa agar. All were Gram-positive, catalase and oxidase negative, non-sporing rods (Supplementary Table S2) and these were further investigated in this study. 16S rDNA sequence analysis showed 55 of the 57 *Lactobacillus* isolates were close relatives of *L. plantarum* (Supplementary Table S3), and two isolates were identified as close relatives of *L. brevis* (Supplementary Table S3). Identification of *L. plantarum* isolates was discriminated from *L. pentosus* and *Lactobacillus paraplantarum* using PCR amplification of *RecA* gene (Supplementary Figure S3). *L. pentosus* formed 1 band of 218 bp, a typed *L. plantarum* NCIMB 318914 formed the band of 318 bp like the Stilton isolates implying similarity. In *L. paraplantarum* the product size is 107 bp which none of our isolates formed. In fact, bands belonging to reference *Lb. pentosus* and those belonging to a single *Lb. plantarum* isolate obtained from Stilton cheese were excised, sequenced and appropriately identified as *recA* gene sequences belonging to *L. pentosus* and *L. plantarum*. The presence of two bands in the multiplex PCR reaction for *Lb. plantarum* isolates obtained from Stilton cheese may be attributed to the fact that these isolates belong to a subspecies of *Lb. plantarum*. There is a need for further studies to assign all *Lactobacillus* isolates to their subspecies.

### PFGE Profiling

**FIGURE 1:**
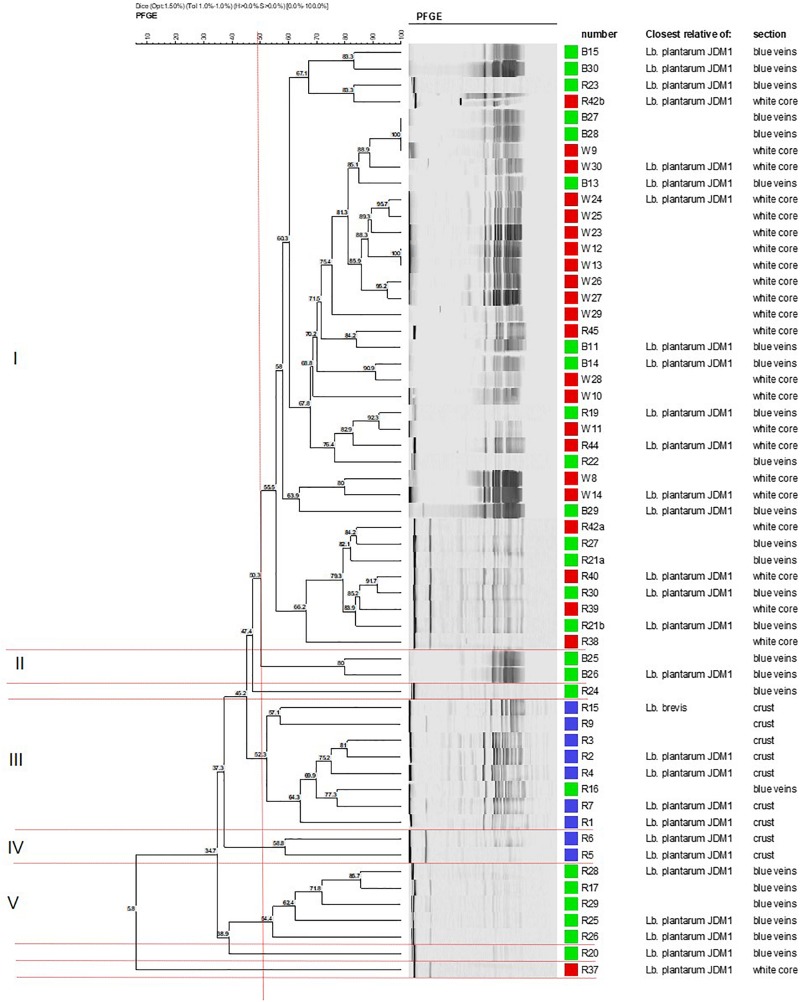
Dendrogram showing the clustering of *Lactobacillus* isolates from different sections in Stilton cheese based on PFGE patterns produced using *Not*1. Similarity values were obtained using the Dice coefficient and clustering by UPGMA. Groups I–V are defined at 52% (vertical line). Strains from: (■) outer crust, (■) blue veins, and (■) white core. All isolates were *Lb. plantarum* except for *Lb. brevis* (strains R9 and R15, cluster III).

Cluster analysis of the 59 identified isolates was conducted to establish the degree of genomic relatedness of the *Lactobacillus* isolates. The dendrogram yielded five major geno-groups at a similarity level of 52% (Φ*_*PT*_* = 0.34; *P* < 0.01; Figure 1). All clusters (except II and IV) had related PFGE profiles and only contained isolates from the blue veins and white core implying clonal relationship. Cluster I was the largest and comprised 37 isolates, whereas clusters II and IV were the smallest and contained two isolates each. *L. plantarum* strains R24 and R20 were outliers to clusters II and V, respectively, while R37 was an outliner to the five clusters. In Group 1, strains B27 and B28 from the blue veins and W9 from the white core showed 100% similarity, as did W12 and W13 from the white core, and so were considered to be identical. All isolates from the outer crust grouped in clusters III and IV with only one blue vein isolate, R16, suggesting they entered into the cheese from a common source. Piercing, which occurs during the ripening process, could have introduced a crust isolate into the veins and could explain the similarity of R16 with this group. This has been suggested to occur in other blue-veined cheeses (Yunita and Dodd, 2018).

Cluster analysis did not delineate *L. brevis* isolates (R9 and R15, Figure 1) as a separate and distinct cluster even though these belong to a different species. The latter were adjacently aligned and grouped with *L. plantarum* in cluster III. This suggests some homology in genomic DNA of these LAB species, although both had been shown to exhibit different API profiles (discussed later).

### Phenotypic Characterization of *Lactobacillus* Isolates

A total of 16 representative *Lactobacillus* isolates (indicated in Figure 1) were characterized on the basis of carbohydrate fermentation patterns (API) and assigned as *L. plantarum* and *L. brevis* in agreement with the 16S rDNA identification. The percent identification for *L. plantarum* isolates ranged from 90.4 to 99.9%, and 95 to 96.1% for *L. brevis* isolates (Supplementary Table S4). *L. plantarum* isolates such as strains R4 and R5, which clustered close to each other, also had similar API profiles, demonstrating good correspondence of the two typing methods. All *Lactobacillus* isolates utilized ribose, glucose and fructose. The fermentation pattern of the other carbohydrates was species-dependent. Both *L. brevis* isolates could only metabolize ribose, xylose, fructose, glucose, and gluconate out of the 50 substrates; *L. brevis* strain R9 could assimilate galactose and α-keto gluconate (profile 6) whereas R15 could assimilate potassium gluconate (profile 7).

Nine of the 14 *L. plantarum* isolates had similar substrate assimilation profiles (profile 4) but four showed a different pattern confirming a mixed population of these organisms. The diversity was caused by differences in the ability to assimilate rhamnose, mannoside, and melezitose. In contrast to all the other *L. plantarum* isolates, strains R4 and R5 (outer crust) and R37 (white core) could assimilate melezitose but not rhamnose; R4 atypically could not assimilate mannoside. The API T-indices were lower than 1.0 suggesting that the profiles were not absolutely typical of the species identified. This also indicates that the API database does not contain a large number of wild isolates demonstrating the diversity of *Lactobacillus* isolates selected in the Stilton sample examined.

### Acid Tolerance

Preliminary experiments on acid stress tolerance of six isolates, two from each cheese section, selected from the three major PFGE clusters (I, III, and V, Figure 1) showed no significant variation in stress response (*p* > 0.05; Mugampoza, 2013). Therefore, three isolates, R2 from the outer crust, B14 from blue veins and W30 from the white core, were selected for further stress tolerance studies using the approach of Catte et al. (1999), and testing for tolerance to heat, acid, salt and desiccation stresses.

**FIGURE 2:**
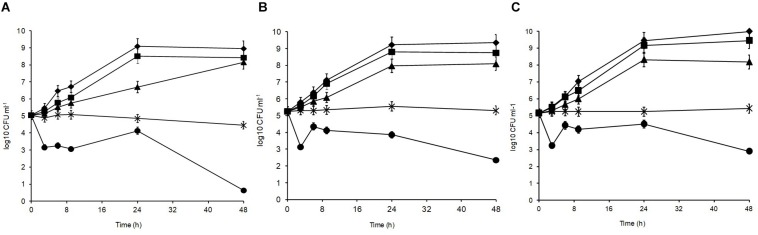
Acid tolerance of *L. plantarum* isolates from Stilton cheese: **(A)** R2, outer crust; **(B)** B14, blue veins; **(C)** W30, white core. Assays were performed in MRS broth at pH: (◆) 6, control, (■) 5, (▲) 4.5, (X) 4, and (🌑) 3.5.

Figure 2 shows the acid tolerance of the three *L. plantarum* isolates in MRS broth acidified to pH 3, 3.5, 4, 4.5, 5, and 6 (control). The viable counts for all isolates were undetectable after 2 h of exposure at pH 3 (data not shown; Mugampoza, 2013). The isolates presented different sensitivities at pH 3.5 leading to gradual cell death. At 48 h, strain R2 from the outer crust (Figure 2A) was the most sensitive (4.4 log_10_ CFU ml^–1^ reduction), whereas B14 from the blue veins (Figure 2B; 2.9 log_10_ CFU ml^–1^ reduction) and W30 from the white core (Figure 2C; 2.3 log_10_ CFU ml^–1^ reduction) were more acid tolerant. Exposure at pH 4 was inhibitory for growth of all the tested isolates, but pH 4.5 and 5 had no significant effect on growth characteristics of the isolates relative to the control (*p* > 0.05). The pH of fresh Stilton curd 2 h after coagulation is approximately 6.7, decreases to approximately 4.8 in a 4 day-old cheese with lower values in the white core, but increases thereafter to approximately 6 at the end of ripening (Madkor et al., 1987).

### Salt Tolerance

**FIGURE 3:**
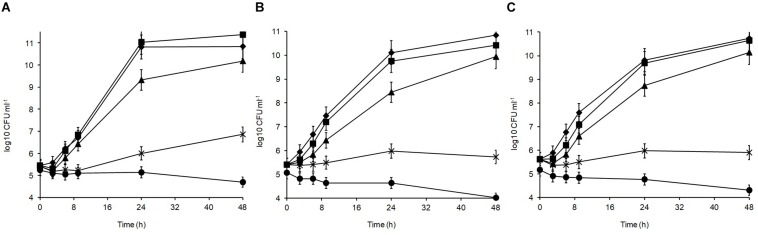
Salt tolerance of *L. plantarum* isolates from Stilton cheese: **(A)** R2, outer crust; **(B)** B14, blue veins; **(C)** W30, white core. Assays were performed in MRS broth supplemented with different salt concentrations (%, w/v): (◆) 0, control, (■) 3.5, (▲) 5, (X) 8, and (🌑) 10.

Different sodium chloride concentrations (3.5, 5, 8, and 10%) in the medium produced differences in the growth patterns of the isolates, with the optimal growth at 0–3.5% (Figure 3). At 48 h, all isolates could grow over the salt range 3.5–5% indicating their high halotolerance. The highest salt concentration (10%) resulted in significant growth suppression (*p* < 0.05) leading to 0.15, 0.86, and 1.05 log_10_ CFU ml^–1^ reductions for *L. plantarum* strains R2, W30, and B14, respectively. At 8%, the isolates from the blue veins (Figure 3B) and white core (Figure 3C) were more sensitive to salt showing only 0.32 and 0.28 log_10_ CFU ml^–1^ increases to final viable counts of 5.7 and 5.9 log_10_ CFU ml^–1^, respectively. In comparison, the isolate from the outer crust (Figure 3A) showed a 1.2 log_10_ CFU ml^–1^ increase to a final population of 6.5 log_10_ CFU ml^–1^.

### Desiccation Tolerance

Desiccation tolerance was performed in SDW in order to avoid salt stress during desiccation. MRD was included to highlight the protective role of peptone for microbial cells subjected to drying conditions. This would allow evaluation of nutrients as a potential risk factor for persistence and subsequent colonization of *Lactobacillus* in the cheese production environment which is the most likely source of cross-contamination onto the outer crust. The isolates varied in sensitivity to initial drying (30°C) prior to desiccation at 20°C, and this depended on the medium in which the cells were suspended (data not shown; Mugampoza, 2013). Suspending cells in MRD caused slight reduction (0.43–0.57 log_10_ CFU ml^–1^) in viable counts whereas in SDW, a higher reduction (0.67–1.13 log_10_ CFU ml^–1^) was observed. In these media, *L. plantarum* strain W30 (white core) was the most tolerant to the initial drying step compared with R2 and B14 from the outer crust and blue veins, respectively (data not shown; Mugampoza, 2013).

**FIGURE 4:**
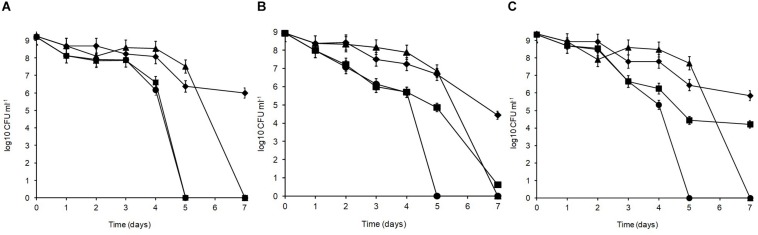
Desiccation tolerance of *L. plantarum* isolates from Stilton cheese: **(A)** R2, outer crust; **(B)** B14, blue veins; **(C)** W30, white core. The cells were exposed at 20°C to different % relative humidity (RH) levels when suspended in maximum recovery diluent (MRD) or sterile distilled water (SDW): (◆) 33% RH in MRD, (▲) 54% RH in MRD, (■) 54% RH in SDW, and (🌑) 33% RH in SDW.

Over 7 days at equilibrated humidity, the sensitivity of the isolates to desiccation stress varied depending on the RH applied and the medium in which the cells were dried (Figure 4). All isolates were sensitive at 33% RH in SDW and became undetectable by 5 days of exposure. All isolates survived desiccation at this RH in MRD over 7 days suggesting cellular protection by MRD. *L. plantarum* strains R2 and W30 showed a 3 log_10_ CFU ml^–1^ reduction over this period whereas B14 showed a further 1 log_10_ CFU ml^–1^ reduction in survival. Survival was higher at 54% RH although after 7 days only W30 showed significant levels of survival in SDW.

In MRD, all isolates were sensitive and died off by 7 days with the greatest inactivation occurring between 5 and 7 days. In SDW, *L. plantarum* R2 from the outer crust (Figure 4A) was more sensitive and survived for only 5 days. The isolate from the blue veins B14 (Figure 4B) showed a low survival level (0.6 log_10_ CFU ml^–1^) by 7 days but W30 from the white core (Figure 4C) showed better tolerance with 4.2 log_10_ CFU ml^–1^ survival up to 7 days. Overall, survival was better at 33% RH in MRD than all other treatments (*p* < 0.05). Therefore, it was concluded that survival of *L. plantarum* is dependent on RH, the drying medium and the strain.

### Heat Tolerance

**FIGURE 5:**
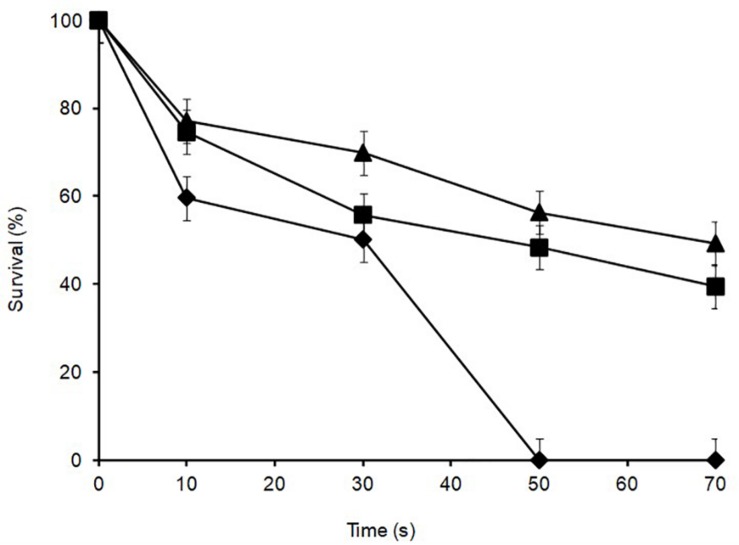
Thermal inactivation kinetics of *L. plantarum* isolates from Stilton cheese heated in milk at 72 ± 1°C: (▲) W30 white core, (■) B14 blue veins, (◆) R2 outer crust. Values are means of two independent determinations and error bars represent ± standard errors. 100% survival corresponds with 8.21, 8.57, and 8.73 log_10_ CFU ml^–1^ for *L. plantarum* R2, B14 and W30, respectively. The come-up time was monitored using a thermocouple in a spare sample tube.

*Lactobacillus plantarum* was heat-treated for 70 s at 72°C in cows’ milk in order to establish their sensitivity during pasteurization. The thermal inactivation of stationary phase *L. plantarum* R2 (outer crust) resulted in undetectable levels after 50 s of heat treatment whereas B14 (blue veins) and W30 (white core) could be recovered after 70 s, although they only grew on the non-selective BHI agar and not the selective MRS agar, which was indicative of heat injury (Figure 5). The differences in *D*_72°C_ values for *L. plantarum* strains R2 (6.9 s, *r*^2^ = 0.92), B14 (20.7 s, *r*^2^ = 0.95), and W30 (23.6 s, *r*^2^ = 0.98) were significant (*p* < 0.05). The greater heat sensitivity of the outer crust isolate is suggestive that this geno-group of *L. plantarum* could have entered into the cheese post-pasteurization, which is also consistent with their location on the surface of the cheese. The calculated *D*_72°C_ values for *L. plantarum* B14 and W30 obtained from the inner cheese matrix (20.7 and 23.6 s, respectively) shows that there would be a less than 1 log_10_ reduction in cell numbers for these isolates during a standard pasteurization over 15 s.

**FIGURE 6:**
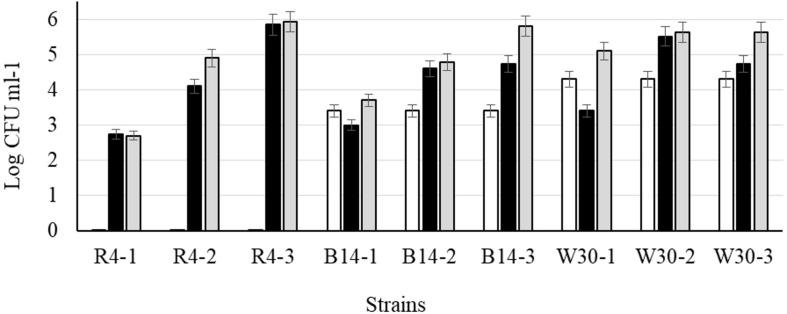
Recovery of stationary phase cells of *L. plantarum* isolates (initial inoculum, 9 log_10_ CFU ml^–1^) heated for 70 s at 72°C and incubated in sterile milk at 4°C for: (□) 0, (■) 24, and (■) 48 h. Values are means of two independent determinations and error bars represent ± standard errors. Strains: R4 (outer crust), B14 (blue veins), W30 (white core); three independent replicates (-1, -2, and -3) were tested for each strain.

After cold storage of the heat-treated samples (4°C, 48 h), part of the cell population recovered from the heat injury as manifested by an increased colony count on the non-selective BHI agar (Figure 6) but not on MRS agar; this is typical of sublethally injured cells being present as part of the bacterial population. On BHI agar, a significant recovery (*p* < 0.05) was observed for *L. plantarum* R2 (outer crust; from <1 to 2.7 log_10_ CFU ml^–1^) and W30 (white core; from 4.3 to 5.1 log_10_ CFU ml^–1^). The increase in cell number was due to recovery and not growth as the corresponding non-heat-treated (control) isolates showed no growth in milk over 48 h at 4°C (Supplementary Figure S1). This was true for cells inoculated at three different count levels (10^7^, 10^4^, and 10^2^ CFU ml^–1^).

### Antimicrobial Activity of Cells

Agar overlays were used to evaluate the spectrum of antimicrobial activity for 50 *L. plantarum* isolates from the different cheese sites (Supplementary Table S4). Each of the *Lactobacillus* isolates showed activity against more than one species but the number of sensitive species varied with the *Lactobacillus* isolate and its site of isolation. All isolates produced inhibitory activity against *L. pentosus*, *Ps. aeruginosa*, *E. coli*, and *L. monocytogenes*, whereas only some showed activity against *Staphylococcus aureus* (92%) and *Salmonella* Typhimurium (96%) and none showed activity against *Clostridium sporogenes*. Of the seven isolates tested from the outer crust, strains R5 and R6 had a weaker activity against *E. coli* and *L. lactis* whereas R1 had no activity against *S. aureus*. *L. plantarum* strains R22, R23, and R37 exerted no activity against *S. aureus*. Similar results were obtained for *L. plantarum* strains B30 and W13 toward *Sal.* Typhimurium. The isolates from the outer crust had the highest level of antimicrobial activity with only *C. sporogenes* and *S. aureus* showing some resistance to these lactobacilli. The data indicated that the isolates could produce different antimicrobials active against a broad range of bacterial species including lactic acid bacteria (LAB) as well as other Gram-positive and Gram-negative organisms. As *L. plantarum* usually produces plantaricins with activity only against closely related LAB (Diep et al., 2009), activity against other bacterial species may be attributed to other mechanisms.

### Antimicrobial Activity of Cell-Free Supernatants

Using the paper diffusion assay, cell-free supernatants of all the 50 *L. plantarum* isolates were screened for antimicrobial activity against *L. pentosus, L. monocytogenes*, *E. coli*, and *Ps. aeruginosa* as these were the most sensitive strains from the agar overlay assay. This protocol allowed for comparison of results obtained using the overlay method and also eliminated the possibility of competition for nutrients through *L. plantarum* growth being an inhibitory factor for the sensitive strains. For all *Lactobacillus* isolates, the cell-free supernatant (CFS) had a narrow spectrum of activity and only showed inhibitory reactions against *E. coli* and *Ps. aeruginosa* (data not shown; Mugampoza, 2013). The CFS could not inhibit the growth of *L. monocytogenes* and only formed a small faint halo regarded as negative for this indicator bacterium. Further examination of the CFS on closely related LAB strains demonstrated that seven (of the 50) isolates had inhibitory activity against *L. pentosus* (Supplementary Table S5), but none was inhibitory against other LAB strains including *L. mesenteroides*, *L. plantarum* NCIMB 138914, *L. lactis*, *Lactobacillus fermentum*, and *Streptococcus thermophilus* (data not shown; Mugampoza, 2013). The CFS with activity against *L. pentosus* was only obtained from *Lactobacillus* isolated from the blue veins and white core. These results suggested that, under our experimental conditions, the CFS of *L. plantarum* isolates could only show antagonism against Gram-negative bacteria and closely related members of the LAB group such as *L. pentosus*. The greatest inhibition, i.e., halos > 2 mm, was obtained when the isolates were grown for 24 h at 30°C at 100 rev min^–1^ in MRS broth supplemented with at least 0.6% glucose. These conditions were then employed to obtain CFS which was treated to neutralize acids and hydrogen peroxide in order to identify the mechanism of activity (Supplementary Table S6).

Cell-free supernatant adjusted to pH 6.5-7 (CFS-N) and catalase-treated CFS (CFS-C) from *L. plantarum* isolates R36, R37, and R38 formed smaller halos (1–2 mm) than the control CFS (2 mm), whereas the corresponding CFS from *L. plantarum* isolates R23, R42, and R45 had halos greater than 2 mm. Reduction in activity when CFS-N and CFS-C were applied suggested that this activity was, in part, due to production of acid and hydrogen peroxide. For all isolates (except R39), catalase-treated CFS adjusted to pH 6.5-7 (CFS-N-C) formed halos of 1–2 mm. A decrease in activity from this treatment was only noted from *L. plantarum* isolates R23 and R42. This indicates that acids and hydrogen peroxide produced by these isolates had similar levels of activity which was reduced upon removal of both antimicrobials. Thus, treated and untreated CFS retained some activity implying bioactive substances, additional to acids and hydrogen peroxide, were produced. CFS treated with the proteolytic enzyme proteinase K lost activity suggesting that a key part of the antagonism was due to a proteinaceous bacteriocin.

### Prevalence and Expression of Bacteriocin Genes

In this work, 54 *L. plantarum* isolates were screened for the presence of plantaricin N, EF, and JK genes using PCR. None of the genes for plantaricins N and JK were detected. However, 35 of the 54 isolates were found to harbor the plantaricin EF genes (data not shown; Mugampoza, 2013). The genes were mostly (6 out of 7) found among the isolates from the outer crust and were least (15 out of 32) frequently detected in isolates from the white core (Figure 1). These findings correlated with the results from the agar overlay assay which showed that *Lactobacillus* isolates from the outer crust had a higher level of antimicrobial activity. All isolates found to contain the plantaricin EF (*plnEF*) genes also expressed some antimicrobial activity, albeit at a lower level. This was the case for *L. plantarum* strains B30, W13 and W30. Presence of the *plnEF* genes showed some correspondence with the data from CFS studies. For instance, neutralized and catalase-treated CFS from isolates that had some activity against *L. pentosus* were also found to harbor this gene, suggesting its expression. However, despite the high prevalence of the gene among the isolates from the outer crust, none of these lactobacilli showed activity in the CFS, implying failure to express the genes.

## Discussion

The identification of bacterial isolates from Stilton revealed the dominance of *L. plantarum* in the LAB species from different cheese sections. Stilton is made from pasteurized milk and should, therefore, be free from non-starter LAB, while *L. lactis*, which is added as starter culture, could not be isolated from the ripened product. This is in agreement with the findings of Ercolini et al. (2003) who reported the presence of this starter culture bacterium in PCR-DGGE profiles from the cheese and from culture plates showing confluent growth but not from plates in the countable range. Cogan et al. (2007) and Hayaloglu (2016) stated that NSLAB identified in most ripened blue cheese varieties made from raw or pasteurized milk are facultative homofermentative lactobacilli that include *Lactobacillus casei, L. plantarum*, and *L. curvatus*, all of which normally grow from levels of 10^2^–10^4^ to ∼10^8^ CFU g^–1^ by the end of ripening. Ares-Yebra et al. (2019) also reported that lactic acid bacteria from a traditional Azorean cheese are dominated by *L. plantarum*, while Dolci et al. (2008) highlighted that *L. plantarum* and *L. paracasei* are the dominant species in ripened Castelmagno cheese. In Stilton, *L. plantarum* could have been introduced into the cheese matrix from various routes: thermoduric strains in milk which survive pasteurization (Briggiler-Marco et al., 2007), strains introduced from equipment during curd milling and cheese piercing prior to packaging, or strains introduced onto the outer crust from the processing plant environment and handlers during ripening.

Biochemical profiles in combination with PFGE patterns revealed variation in the sugar utilization patterns of the isolates belonging to the same species, implying the presence of different geno-groups of these organisms within each of the cheese sites. Although PFGE clustered *L. brevis* with *L. plantarum* in cluster III, suggesting low compatibility of the methodologies, phenotypic characteristics were congruent with the genotyping data including PFGE profiling and 16S rDNA sequence analysis (Mugampoza, 2013, Appendix 8).

Given that PFGE grouping generated at 52% similarity (Tenover et al., 1995) was based on site of isolation (crust, core, and blue veins) and since preliminary experiments revealed little variation in growth characteristics of *L. plantarum* isolates obtained from the same cheese site (data not shown), only isolates coming from different PFGE clusters and cheese sites were tested for stress tolerance. As the selected isolates (R2, outer crust; B14, blue veins; and W30, white core) had faster growth in milk and had different biochemical profiles measured using API, it was anticipated that these isolates, could also show phenotypic differences including stress tolerance and other technological aspects. Indeed, the results in Figures 2–5 revealed diversity in *Lactobacillus* from a single Stilton cheese.

*Lactobacillus plantarum* isolates evidenced good survival at different levels of acid, salt, relative humidity, and heat treatment showing that they can have optimal adaptability in the cheese microenvironment where conditions are normally unfavorable for microbial growth. The data highlighted that *Lactobacillus* in a Stilton cheese represents a mixed species with strains at different sites selected on the basis of their stress tolerances. Randazzo et al. (2002); Duthoit et al. (2003) and Sawitzki et al. (2009) proposed that occurrence, in cheese, of multiple strains of LAB belonging to the same species is, in most cases, the result of ecological selection following introduction into the cheese which may explain our data. Thus, strains in the core which showed greater heat resistance may have come from the original milk and survived pasteurization; these were also more acid tolerant and therefore able to survive the lower pH found in the cheese core. In contrast those on the outer crust were more heat sensitive and could have been introduced post-pasteurization onto the surface by handling or from environmental sources. These latter were more desiccation tolerant and thus show greater ability to be retained in the production environment. Similar studies were performed by Welsh (2000) on *Rhizobium* spp., Succi et al. (2005) on *Lactobacillus rhamnosus* and Pelinescu et al. (2009) on *Lactobacillus* and *Enterococcus* strains. All these authors reported optimal survival following exposure to stress conditions relevant to the site of isolation. In all cases, long-term exposure to stronger stresses caused significant loss of survival which is similar to the findings in this study.

Lund et al. (2000) critiqued the different approaches to determine *D*-values in milk for *Mycobacterium paratuberculosis* and pointed out that there is no best approach to estimate *D*-values in milk simulating the effects of a commercial pasteuriser. Thus, the submerged tube method used in this study would not relate the *D*-values obtained with industrial process, but rather used to determine the relative heat sensitivity of different *L. plantarum* isolates. However, there is a need to overcome the problem of come-up time during heat stress studies which could have affected results in our experimental model. Further research in preheated milk, or use of sealed capillary tubes could be employed (Chung et al., 2007).

Some of the *L. plantarum* isolates could produce the heat-stable plantaricin EF bacteriocins. The bacteriocins were only active against *L. pentosus*. The result was in agreement with the relatively narrow inhibitory spectrum reported for plantaricins (Nissen-Meyer et al., 1993; Diep et al., 2009), mostly being active against bacterial species closely related to the producer *L. plantarum* strains. However, most of the isolates had a broad spectrum of antimicrobial activity against the Gram-negative and Gram-positive bacteria on solid medium. Presence of the *plnEF* genes showed no association with the PFGE clusters (Supplementary Figure S2). For instance, cluster I contained the highest number of genetically similar *L. plantarum* isolates suggesting they were clones of a single strain. However, *plnEF* data highlighted some variations within this cluster; even closely related isolates within sub-clusters obtained from the same site gave different results. For example, in cluster I, the first sub-cluster of four isolates which formed at a similarity level of 67% contained the isolates B15/B30 which were negative for *plnEF* and R23/R42b which were positive for these genes. However, identical genotypes gave the same *plnEF* gene results as evident for isolates B27/B28/W9 and W12/W13. Thus, a PFGE geno-group did not define a single identical strain. Therefore, it was concluded that there were different strains or sub-populations of *L. plantarum* in the Stilton sample examined. Some strains were found at more than one site but others were site-specific which was in agreement with previous studies on the diversity of *Lactobacillus* strains (Zapparoli et al., 1998; Girrafa and Neviani, 1999; Sanchez et al., 2004; Vancanneyt et al., 2006). Future research may employ whole genome sequencing and/or multi-locus sequence typing (MLST) to provide more insight about the clonal relationship between *Lactobacillus* isolates used in this study with those of other authors.

## Conclusion

The dynamic and complex microenvironments in different sites of Stilton cheese seem to play a major role in selecting for different strains of *Lactobacillus*. Because these organisms are fortuitously introduced into Stilton cheese, this could be a source of product quality inconsistencies as pointed out by Montel et al. (2014). Whereas the drawbacks of NSLAB as culture adjuncts in cheese were pointed out by Gobbetti et al. (2015), the observed properties of *L. plantarum* isolates could be of interest in developing *Lactobacillus* starters and starter culture adjuncts with improved resistance to multiple stresses for several applications including probiotics, where growth under harsh stress conditions would be an attribute. Use of *L. plantarum* as a starter culture adjunct for Stilton cheese would need to take the varying phenotypes into consideration.

However, our results were obtained at laboratory scale from a single cheese taken at a single time point and from a single source. Transcriptome and proteome studies coupled with microarray analysis may be applied to validate whether or not stress tolerance of our isolates could be triggered by transient exposure to a wide range of sub-lethal factors including acid, salt, drying and oxidative stress at different gradients within the cheese matrix. Elucidation of these mechanisms would add to the understanding of how the organism survives the cheese ripening process and would enable a more targeted approach to non-starter culture adjunct selection for quality improvement, maintenance and reliability of Stilton cheese production. As the primer set used for 16S rRNA gene sequencing of our *Lactobacillus* isolates was short (448 bp), further research could employ a primer set that covers the complete 16S gene to generate more stringent identification of the lactobacilli.

## Data Availability Statement

All datasets generated for this study are included in the article/Supplementary Material.

## Author Contributions

The study was conceived and designed by DM, KG, and CD who all contributed to acquisition, analysis, and interpretation of the data and writing of the manuscript. BS and CR devised the methodology for the heat inactivation and recovery experiments. All authors approved the final version of the manuscript.

## Conflict of Interest

The authors declare that the research was conducted in the absence of any commercial or financial relationships that could be construed as a potential conflict of interest.
