# Supplementary material for: Diversity of Lactobacillus Species of Stilton Cheese Relates to Site of Isolation
Source: Front Microbiol. 2020 May 12;11:904. doi: 10.3389/fmicb.2020.00904 (PMC7236593; doi:10.3389/fmicb.2020.00904)
Supplement: Supplementary file 1 [file Data_Sheet_1.pdf]

Table S1 List of bacterial isolates obtained from different sites of Stilton cheese and their NCBI BLAST match identifications

S/No	Isolate	Site	Close relative of
1	R1	crust	*Lb. plantarum*
2	R2	crust	*Lb. plantarum*
3	R3	crust	*Lb. plantarum*
4	R4	crust	*Lb. plantarum*
5	R5	crust	*Lb. plantarum*
6	R6	crust	*Lb. plantarum*
7	R7	crust	*Lb. plantarum*
8	R16	veins	*Lb. plantarum*
9	R17	veins	*Lb. plantarum*
10	R19	veins	*Lb. plantarum*
11	R20	veins	*Lb. plantarum*
12	R21a	veins	*Lb. plantarum*
13	R21b	veins	*Lb. plantarum*
14	R22	veins	*Lb. plantarum*
15	R23	veins	*Lb. plantarum*
16	R24	veins	*Lb. plantarum*
17	R25	veins	*Lb. plantarum*
18	R26	veins	*Lb. plantarum*
19	R27	veins	*Lb. plantarum*
20	R28	veins	*Lb. plantarum*
21	R29	veins	*Lb. plantarum*
22	R30	veins	*Lb. plantarum*
23	B11	veins	*Lb. plantarum*
24	B13	veins	*Lb. plantarum*
25	B14	veins	*Lb. plantarum*

26	B15	veins	*Lb. plantarum*
27	B23	veins	*Lb. plantarum*
28	B24	veins	*Lb. plantarum*
29	B25	veins	*Lb. plantarum*
30	B26	veins	*Lb. plantarum*
31	B27	veins	*Lb. plantarum*
32	B28	veins	*Lb. plantarum*
33	B29	veins	*Lb. plantarum*
34	B30	veins	*Lb. plantarum*
35	R36	core	*Lb. plantarum*
36	R37	core	*Lb. plantarum*
37	R38	core	*Lb. plantarum*
38	R39	core	*Lb. plantarum*
39	R40	core	*Lb. plantarum*
40	R42a	core	*Lb. plantarum*
41	R42b	core	*Lb. plantarum*
42	R44	core	*Lb. plantarum*
43	R45	core	*Lb. plantarum*
44	W8	core	*Lb. plantarum*
45	W9	core	*Lb. plantarum*
46	W10	core	*Lb. plantarum*
47	W11	core	*Lb. plantarum*
48	W12	core	*Lb. plantarum*
49	W13	core	*Lb. plantarum*
50	W14	core	*Lb. plantarum*
51	W15	core	*Lb. plantarum*
52	W23	core	*Lb. plantarum*
53	W24	core	*Lb. plantarum*

54	W25	core	*Lb. plantarum*
55	W26	core	*Lb. plantarum*
56	W27	core	*Lb. plantarum*
57	W28	core	*Lb. plantarum*
58	W29	core	*Lb. plantarum*
59	W30	core	*Lb. plantarum*
60	R9	crust	*Lb. brevis*
61	R15	crust	*Lb. brevis*
62	W22	core	*Acinetobacter baumanii*
63	Ou9	crust	*Enterococcus faecalis*
64	Ou8	crust	*Staphylococcu aureus*
65	Ou10	crust	*Staph. aureus*
66	Ou21	crust	*Staph. aureus*
67	Ou25	crust	*Staph. aureus*
68	Ou30	crust	*Staph. aureus*
69	Ou6	crust	*Psychrobacter cryohalolentis*
70	Ou7	crust	*Ps. cryohalolentis*
71	Ou8	crust	*Ps. cryohalolentis*
72	Ou11	crust	*Ps. cryohalolentis*
73	Ou12	crust	*Ps. cryohalolentis*
74	Ou14	crust	*Ps. cryohalolentis*
75	Ou15	crust	*Ps. cryohalolentis*
76	Ou22	crust	*Ps. cryohalolentis*
77	Ou23	crust	*Ps. cryohalolentis*
78	Ou26	crust	*Ps. cryohalolentis*
79	Ou28	crust	*Ps. cryohalolentis*
80	Ou29	crust	*Ps. cryohalolentis*

Table S2 Phenotypic and biochemical characteristics of bacterial groups isolated from Stilton cheese

Group	Site of isolation	Catalase	Oxidase	Gram stain	Cell morphology	Number (%) of isolates
1	Outer crust	-	-	+	cocci	1 (1.25)
2	Outer crust	+	-	+	cocci	5 (6.25)
3	Outer crust	+	+	-	cocco-bacillus	12 (15.0)
4	White core	+	-	-	cocco-bacillus	1 (1.25)
5	Outer crust, blue veins, white core	-	-	+	rods	61 (76.25)

Table S3 BLAST matches of the sequences from PCR amplification of genomic DNA from *Group 1-5* isolates. Identification was based on variable V3 and V6-V8 regions of 16S rDNA

Isolates	Primer	Amplicon size (bp)	Closest relative	% ID	E-value	Matching sequence length (nt)	Genbank accession number
Group 1	V3	200	*En. faecalis*	99	2^{-47}	111	NC004668
Group 2	V3	200	*Staph. aureus*	97	8^{-46} - 4^{-65}	155-157*	NC013450
Group 3	V3	200	*Ps. cryohalolentis*	95-96*	2^{-57} - 8^{-57}	94-145*	NC007969
Group 4	V3	200	*Ac. baumanii*	95	4^{-55}	144	NC011586
Group 5a	V6-V8	428	*Lb. plantarum*	99-100*	3^{-72} - 4^{-156}	165-311*	NC012984
Group 5b	V6-V8	428	*Lb. brevis*	99-100*	2^{-41} - 1^{-112}	98-231*	NC008497

*Results for a range of isolates: *Group 2*, 3 isolates; *Group 3*, 7 isolates; and *Group 5*, 32 isolates.

Table S4 Substrate assimilation profiles of *Lactobacillus* isolated from Stilton cheese. Data obtained from two independent determinations

Substrate	*L. brevis*		*L. plantarum*						
	R9	R15	R2	R4	R5	B14	R17	R36	R37
D-ribose	+	+	+	+	+	+	+	+	+
D-xylose	+	+	-	-	-	-	-	-	-
D-galactose	+	-	+	+	+	+	+	+	+

D-glucose	+	+	+	+	+	+	+	+	+
D-fructose	+	+	+	+	+	+	+	+	+
D-mannose	-	-	+	+	+	+	+	+	+
L-rhamnose	-	-	+	-	-	+	+	+	-
D-mannitol	-	-	+	+	+	+	+	+	+
D-sorbitol	-	-	+	+	+	+	+	+	+
Methyl-αD-mannoside	-	-	+	-	+	+	+	+	+
N-acetyl glucosamine	-	-	+	+	+	+	+	+	+
Amygdalin	-	-	+	+	+	+	+	+	+
Arbutin	-	-	+	+	+	+	+	+	+
Esculin/ferric citrate	-	-	+	+	+	+	+	+	+
Salicin	-	-	+	+	+	+	+	+	+
D-cellobiose	-	-	+	+	+	+	+	+	+
D-maltose	-	-	+	+	+	+	+	+	+
D-lactose	-	-	+	+	+	+	+	+	+
D-sucrose	-	-	+	+	+	+	+	+	+
D-trehalose	-	-	+	+	+	+	+	+	+
D-melezitose	-	-	-	+	+	-	-	-	+

Gentiobiose	-	-	+	+	+	+	+	+	+
D-arabitol	-	-	+	+	+	+	-	+	+
Potassium gluconate	+	+	-	-	-	-	-	-	-
α-keto-gluconate	+	-	-	-	-	-	-	-	-
%ID (API)	95	96.1	99.9	90.4	99.8	99.9	99.9	99.9	99.9
API t-index	0.47	0.7	0.6	0.72	0.6	0.6	0.74	0.6	0.73
%ID (16S rDNA)	96	96	100	99	98	99	99	99	99

(+) positive reaction, (-) negative reaction, ND (not done). Isolates were obtained from the: outer crust (R2-R15), blue veins (B14, R17), and white core (R36, R37)

Table S5 Proportion of *L. plantarum* isolates that demonstrated inhibitory activity against the various indicator bacteria based on agar plate overlay assay

Indicator strain	*% *L. plantarum* strains inhibitory against the indicator strains			
	Crust (n=7)	Veins (n=19)	Core (n=24)	(N=50)
Listeria monocytogenes NCTC 11944	100	100	100	100
Staph. aureus (Stilton isolate)	86	89	96	92
Staph. aureus NCTC 12100	100	95	96	96
E. coli 0157: H7-stx	100	100	100	100
Salmonella. Typhimurium	100	95	96	96

Ps. aeruginosa glaxo-3	100	100	100	100
C. sporogenes	000	000	000	000
Lactococcus lactis NCIMB 9918	100	100	24	100
L. pentosus NCIMB 8026	100	100	100	100

*Based on the presence (+) or absence (-) of a halo around the colonies of LAB strains

Table S6 Antimicrobial activity of treated and untreated cell-free supernatants of *L. plantarum* isolates against *L. pentosus,* and *Pediococcus acidilactici* (pediocin-producing control strain) against *Listeria monocytogenes*

Extract		*L. plantarum* isolates						
		blue veins	white core					
	P. acidilactici[*]	R23[**]	R36[**]	R37[**]	R38[**]	R39[**]	R42[**]	R45[**]
CFS	+++	+++	+++	++	+++	+++	+++	+++
CFS-N	+++	+++	++	++	++	++	+++	++
CFS-C	+++	+++	++	++	++	++	+++	+++
CFS-N-C	+++	++	++	++	++	+++	++	++
CFS-P	-	-	-	-	-	-	-	-

CFS, cell free supernatant; CFS-N, CFS neutralized to pH 7; CFS-C, CFS catalase-treated to remove H_2O_2; CFS-N-C, CFS neutralized and catalase-treated; CFS-P, CFS protease treated.

(-): no zone of inhibition was observed or a zone less than 0.5mm in diameter.

(+): a zone of inhibition greater than or equal to 0.5mm but less than or equal to 1 mm in diameter.

(++): a zone of inhibition greater than 1 mm and less than or equal to 2 mm in diameter.

(+++): a zone of inhibition greater than 2 mm in diameter

* tested against *Listeria monocytogenes*

** tested against *L. pentosus*
