# Supplementary material for: Diversity of Lactobacillus Species of Stilton Cheese Relates to Site of Isolation
Source: Front Microbiol. 2020 May 12;11:904. doi: 10.3389/fmicb.2020.00904 (PMC7236593; doi:10.3389/fmicb.2020.00904)
Supplement: Supplementary file 2 [file Data_Sheet_2.pdf]

**SUPPORTING INFORMATION**

# Diversity of *Lactobacillus* Flora of Stilton Cheese relates to Site of Isolation

***Diriisa Mugampoza[1,2*], Konstantinos Gkatzionis[1,3], Benjamin M.C. Swift[1,4], Catherine E.D. Rees[1] and Christine E.R. Dodd[1]***

[1]Division of Food Sciences, University of Nottingham, Leicestershire, United Kingdom

[2]Department of Food Technology, Kyambogo University, Kampala, Uganda

[3]Department of Food Science and Nutrition, School of the Environment, University of the Aegean, Metropolite Ioakeim 2, GR 81400, Myrina, Lemnos, Greece

[4]Pathobiology and Population Sciences, Royal Veterinary College, Hertfordshire, United Kingdom

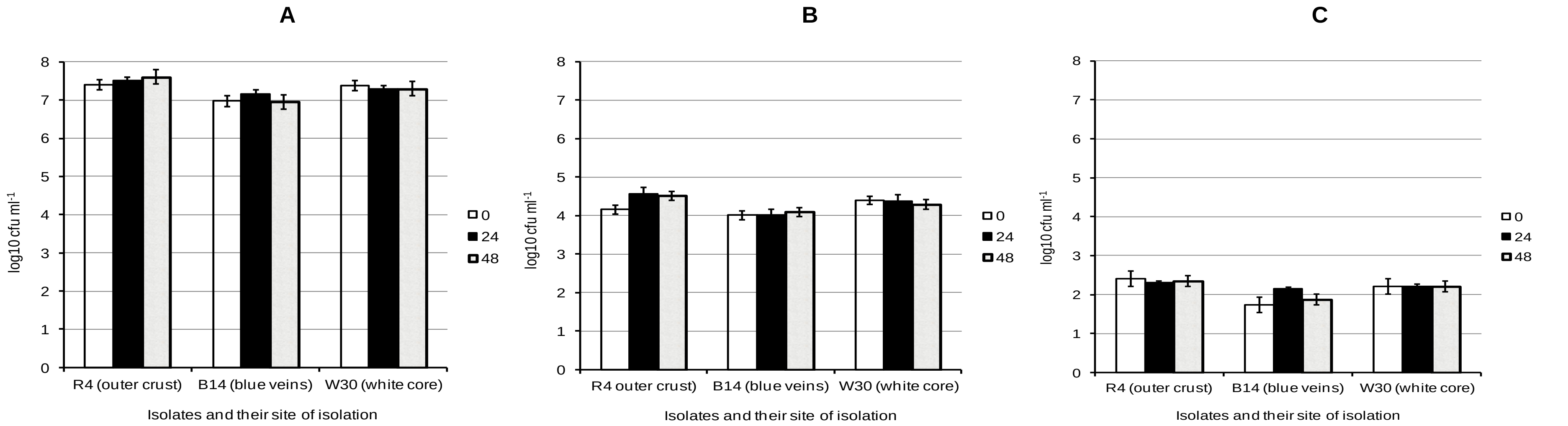


**Figure S1** Viable counts (log$_{10}$ cfu ml$^{-1}$) of stationary phase cells of *L. plantarum* R2 (outer crust), B14 (blue veins) and W30 (white core) in cows' milk at 4$^{o}$C after incubating for: (□) 0, (■) 24, and (■) 48 h. Counts were taken from BHI agar after incubating anaerobically for 48 h at 30$^{o}$C. Values are means of two independent determinations and error bars are ± standard errors of the means. The milk was spiked with a final cell concentration of: (**A**) 10$^{7}$, (**B**) 10$^{4}$, and (**C**) 10$^{2}$ CFU ml$^{-1}$.

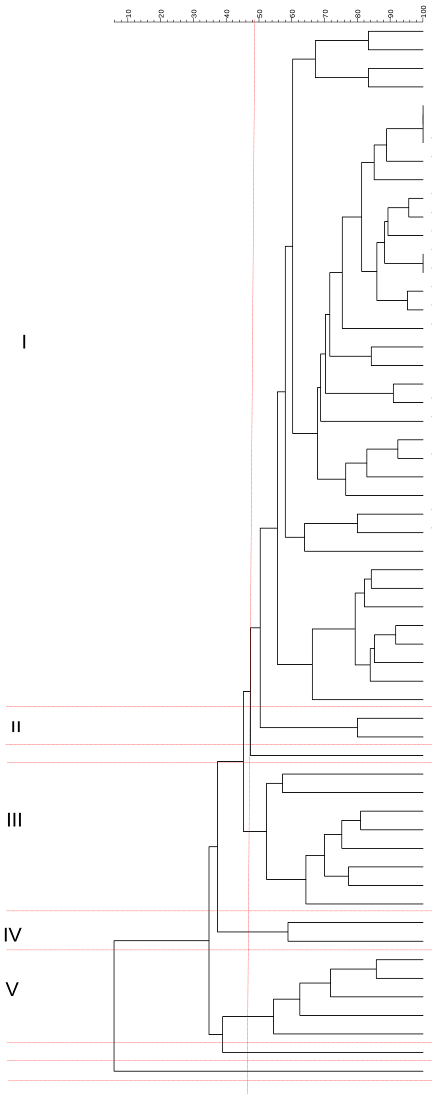


| Isolates | Close relative | Isolation site | PlnEF gene | API profiles |
|---|---|---|---|---|
| B15 | Lact. plantarum | blue veins | - | ND |
| B30 | Lact. plantarum | blue veins | - | ND |
| R23 | Lact. plantarum | blue veins | + | 4 (melezitose) |
| R42b | Lact. plantarum | white core | + | ND |
| B27 | | blue veins | + | 4 (melezitose) |
| B28 | | blue veins | + | ND |
| W9 | | white core | + | ND |
| W30 | Lact. plantarum | white core | - | ND |
| B13 | Lact. plantarum | blue veins | + | ND |
| W24 | Lact. plantarum | white core | + | 4 (melezitose) |
| W25 | | white core | + | ND |
| W23 | | white core | - | ND |
| W12 | | white core | - | ND |
| W13 | | white core | - | ND |
| W26 | | white core | - | 4 (melezitose) |
| W27 | | white core | - | ND |
| W29 | | white core | - | ND |
| R45 | | white core | + | ND |
| B11 | Lact. plantarum | blue veins | + | ND |
| B14 | Lact. plantarum | blue veins | - | 4 (melezitose) |
| W28 | | white core | - | ND |
| W10 | | white core | + | 4 (melezitose) |
| R19 | Lact. plantarum | blue veins | + | ND |
| W11 | | white core | + | ND |
| R44 | Lact. plantarum | white core | + | 4 (melezitose) |
| R22 | | blue veins | + | ND |
| W8 | | white core | + | ND |
| W14 | Lact. plantarum | white core | + | ND |
| B29 | Lact. plantarum | blue veins | + | ND |
| R42a | | white core | + | ND |
| R27 | | blue veins | + | ND |
| R21a | | blue veins | + | ND |
| R40 | Lact. plantarum | white core | + | 4 (melezitose) |
| R30 | Lact. plantarum | blue veins | + | 4 (melezitose) |
| R39 | | white core | + | ND |
| R21b | Lact. plantarum | blue veins | + | ND |
| R38 | | white core | + | ND |
| B25 | | blue veins | + | ND |
| B26 | Lact. plantarum | blue veins | + | ND |
| R24 | | blue veins | + | ND |
| R15 | Lact. brevis | outer crust | - | 7 (galactose, brevis) |
| R9 | | outer crust | - | 6 (gluconate, brevis) |
| R3 | | outer crust | - | ND |
| R2 | Lact. plantarum | outer crust | + 4 | 1 (melezitose & gluconate) |
| R4 | Lact. plantarum | outer crust | + | 2 (rhamnose & mannoside) |
| R16 | | blue veins | + | ND |
| R7 | Lact. plantarum | outer crust | + | ND |
| R1 | Lact. plantarum | outer crust | + | ND |
| R6 | Lact. plantarum | outer crust | + | ND |
| R5 | Lact. plantarum | outer crust | + | 3 (rhamnose) |
| R28 | Lact. plantarum | blue veins | + | ND |
| R17 | | blue veins | - | 5 (arabitol) |
| R29 | | blue veins | - | ND |
| R25 | Lact. plantarum | blue veins | + | ND |
| R26 | Lact. plantarum | blue veins | + | ND |
| R20 | Lact. plantarum | blue veins | + | ND |
| R37 | Lact. plantarum | white core | + | 3 |

**Figure 1**

**Figure S2** Dendrogram showing the clustering of 59 *Lactobacillus* isolates obtained from Stilton cheese. The figure includes corresponding data on site of isolation and presence/absence of the plantaricin EF genes. Similarity values were obtained by UPGMA and the Dice coefficient methods with a 1.5% band position tolerance.

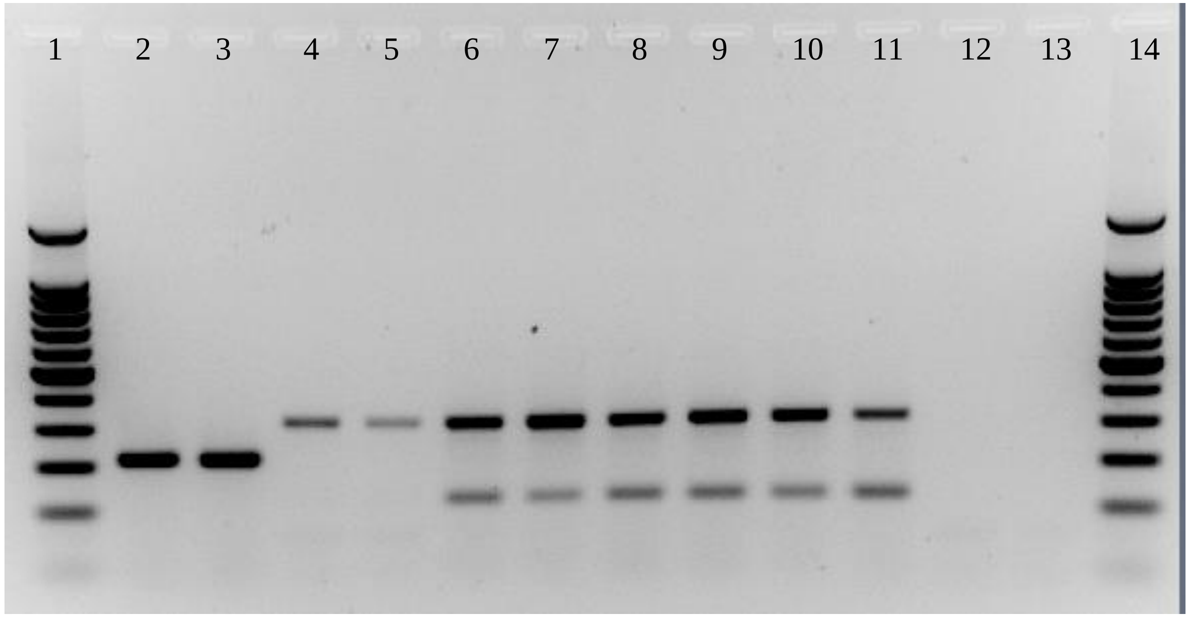


**Figure S3** Rec A gene multiplex PCR analysis of *Lactobacillus plantarum* isolates obtained from Stilton cheese. Lanes 1&14, 100 bp marker; lanes 2&3, *Lb. pentosus* NCIMB 8026; lanes 4&5, *Lb. plantarum* NCIMB 318914; lanes 6-11, *Lb. plantarum* isolates from Stilton cheese: R2 & R4 (outer crust), B13 & B30 (blue veins), and R44 and W30 (white core); lanes 12&13, *Lb. brevis* isolate from Stilton cheese (negative control). The samples were run on 2% (w/v) agarose gel in 1X TAE buffer for 2 h at 70 V.
